# Roles of GATA6 during Gonadal Development in Japanese Flounder: Gonadogenesis, Regulation of Gender-Related Genes, Estrogen Formation and Gonadal Function Maintenance

**DOI:** 10.3390/ijms18010160

**Published:** 2017-01-16

**Authors:** Zan Li, Xiumei Liu, Yan Sun, Jinxiang Liu, Yuezhong Liu, Mengxun Wang, Quanqi Zhang, Xubo Wang

**Affiliations:** Ministry of Education Key Laboratory of Marine Genetics and Breeding, College of Marine Life Sciences, Ocean University of China, Qingdao 266003, China; lizanlxm@163.com (Z.L.); xiumei0210@163.com (X.L.); sy18306421070@163.com (Y.S.); ljkmn911@outlook.com (J.L.); yuezhong_liu@163.com (Y.L.); wang_mengxun@163.com (M.W.); qzhang@ouc.edu.cn (Q.Z.)

**Keywords:** *Paralichthys olivaceus*, GATA6, dimorphic expression, hormone stimulation, gonadal development

## Abstract

GATA-binding protein 6 (GATA6), a highly-conserved transcription factor of the GATA family plays an important role in gonadal cell proliferation, differentiation and endoderm development. In this study, the full-length cDNA of GATA6 of *Paralichthys olivaceus* (Japanese flounder) was obtained. Phylogenetic, gene structure and synteny analyses demonstrated that GATA6 of *P. olivaceus* is homologous to that of teleosts and tetrapods. The *P. olivaceus* GATA6 transcript showed higher expression in testis than in ovary, demonstrating a sexually dimorphic gene expression. During embryonic development, the expression of *P. olivaceus* GATA6 increased at the blastula stage, demonstrating that GATA6 is involved in morphogenesis. Results of in situ hybridization showed that GATA6 signals were detected in Sertoli cells, oogonia and oocytes. Moreover, 17α methyl testosterone, a male hormone, could moderately upregulate *P. olivaceus* GATA6 and downregulate *P. olivaceus* aromatase CYP19A1 in testis cells. These results suggest that GATA6 may play an important role in gonadal development in *P. olivaceus*. This study provides valuable information on the function of *P. olivaceus* GATA6, laying the foundation for further development of breeding techniques in this species.

## 1. Introduction

GATA-binding proteins (GATA1-GATA6) are a family of zinc finger transcription factors that regulate the expression of target genes by binding to a common sequence, namely WGATAR. GATA transcription factors play essential roles in the developmental control of cell fate, proliferation and differentiation, organ morphogenesis and tissue-specific gene expression. These proteins, which were identified in vertebrates and invertebrates, are evolutionarily conserved [1,2,3]. All GATA proteins bear two conserved N- and C-terminal zinc finger domains. The C-terminal zinc finger domain is required for binding, whereas the N-terminal zinc finger can stabilize binding and interaction with other cofactors. Based on spatial and temporal expression patterns, GATA proteins can be categorized into two subgroups, GATA1/2/3 and GATA4/5/6. GATA1/2/3 are mainly found in hematopoietic cell lineages. They are essential in the differentiation of erythroids and megakaryocytes, the proliferation of hematopoietic stem cells and the development of T lymphocytes [4]. By contrast, GATA4/5/6 are found in the organs of mesodermal and endodermal origin, such as heart, gut and gonads [5,6,7,8].

Being a member of the GATA family, GATA6 plays important roles in various development processes, such as cardiac development [6]. GATA6 was recently found to induce a reprogramming process and can be a substitute for Oct4 [9]. To date, many studies have shown that GATA6 is involved in reproduction and gonadal development. The role of GATA6 in gonadal development in human and mouse is well known. *GATA6* mRNA is found in both granulosa cells and corpora luteum, and it regulates the differentiation and proliferation of granulosa cells [10,11]. Knockdown of GATA4 and GATA6 during luteinization inhibits progesterone production and gonadotropin responsiveness in corpus luteum in mice [12]. *GATA6* mRNA and protein are expressed in Sertoli and Leydig cells during early stages of gestation [13]. In addition, both GATA4 and GATA6 transcripts were observed in late fetal, neonatal, juvenile and adult Sertoli cells in mouse testis. In female mice, the loss of GATA4 and GATA6 resulted in failed ovulation and infertility [14]. The combined loss of GATA4 and GATA6 could cause abnormalities in testis, including irregular testis cords, few gonocytes and loss of steroidogenic testis function [15]. Additionally, lines of evidence have shown that some target genes of GATA6 in endocrine tissues play important roles in gonadal development and sex differentiation; these genes include *Amh*, *CYP11A* and *CYP17* [16,17]. Little is known about *GATA6* expression and function in teleosts. In *Cynoglossus semilaevis*, *GATA6* expression is sexually dimorphic, and the methylation pattern in the promoter region varied among males, females and pseudomales [18].

*P. olivaceus* is an economically important farmed marine fish in China. In this species, females exhibit higher growth rates than males, and they attain larger sizes. Therefore, understanding the regulation of sex differentiation and sex-controlled breeding in this species is both scientifically and commercially necessary. In this study, we obtained the full-length of GATA6 from *P. olivaceus* and assessed its relative expression pattern in different tissues and in the early stages of embryonic development. We analyzed *GATA6* expression levels in ovary and testis using in situ hybridization (ISH). Moreover, *P. olivaceus* testis cells were treated with hormone. Our results will facilitate further studies on the function of GATA6 in teleosts and provide fundamental information for sex-controlled breeding techniques.

## 2. Results

### 2.1. Isolation and Characterization of GATA6 cDNA

**Figure 1:**
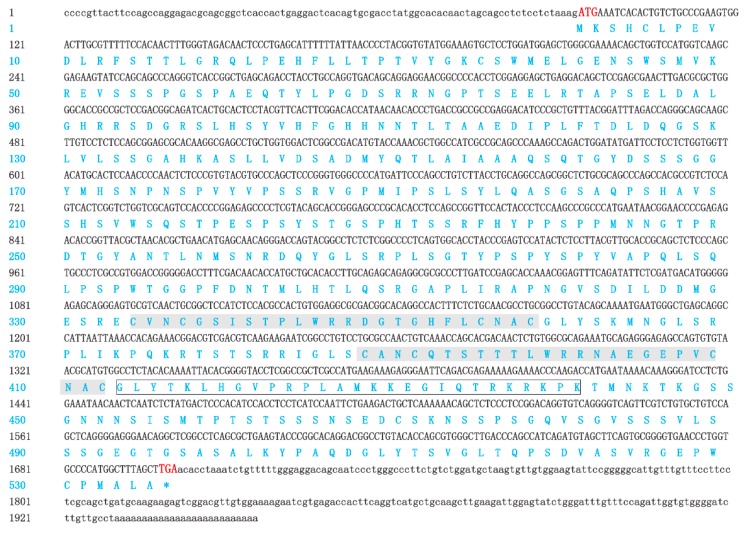
Nucleotide and deduced amino acid sequences of *P. olivaceus* GATA6. Nucleotides are indicated in black and the amino acids in blue. Initiation codon and termination codon are in red. UTRs (Untranslated Regions) and ORF (Open Reading Frame) are denoted in lowercase and uppercase letters, respectively. The zinc finger domains are shaded, whereas the nuclear localization sequence is boxed.

**Figure 2:**
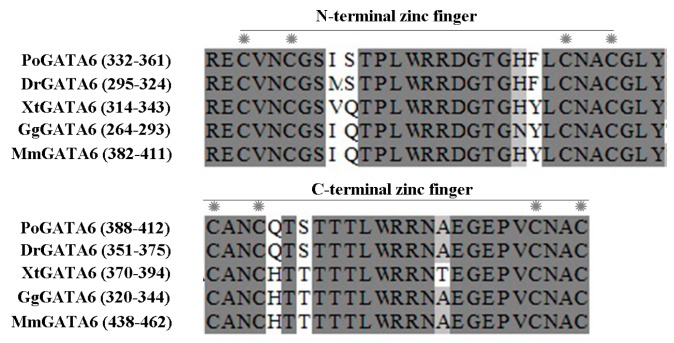
Amino acid sequence alignment of zinc finger domains of PoGATA6, DrGATA6, XtGATA6, GgGATA6 and MmGATA6. Asterisks show the cysteine that generates each zinc finger domain. Abbreviations: Po, *P. olivaceus*; Dr, *D. rerio*; Xt, *X. tropicalis*; Gg, *G. gallus*; Mm, *M. musculus*.

**Figure 3:**
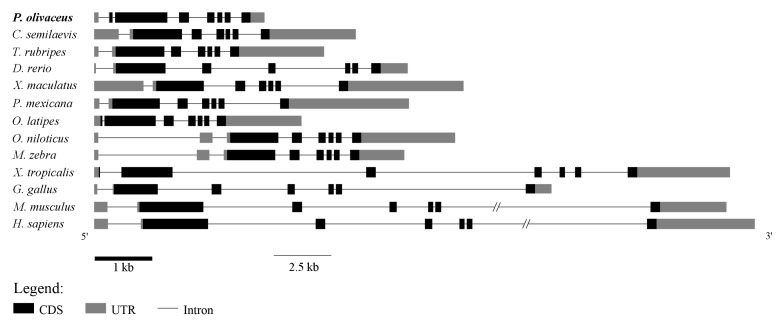
Gene structure analysis of GATA6. Comparisons of genomic organizations of GATA6 between teleosts and tetrapods. CDS (Coding Sequence) and UTRs are indicated by black and gray boxes, respectively, whereas introns are represented by lines.

The complete cDNA sequence of GATA6 was obtained using RACE. The full-length cDNA of GATA6 was 1957 bp long (GenBank: KY039315) and consisted of a 92-bp 5′-UTR (Untranslated Regions), a 257-bp 3′-UTR with a poly(A) tail and an ORF (Open Reading Frame) of 1608 bp (Figure 1). The ORF encoded a polypeptide comprising 535 amino acids with a predicted molecular weight of 57.28 kDa and a theoretical isoelectric point of 8.82. Similar to that of other species, the predicted peptide of GATA6 contained two conserved GATA-type zinc finger domains (amino acid Nos. 332–361, Nos. 388–412) (Figure 1 and Figure 2). The C-terminal zinc finger of GATA is responsible for binding to the consensus DNA sequence WGATAR, whereas the N-terminal zinc finger interacts with transcription cofactors and stabilizes the binding [1,19]. Hence, the obtained sequence was confirmed to be *P. olivaceus GATA6*. Comparison of cDNA and genomic sequences showed that *GATA6* contains eight exons (Figure 3), which differed from most other known species. Based on the gene structure analysis, the 5′-UTR sequences of *GATA6* were located on the first exon and in a portion of the second exon (Figure 3).

### 2.2. Homology and Phylogenetic Analysis

Alignment of deduced amino acid sequences of *P. olivaceus* GATA6 with those of other vertebrates showed that GATA6 protein of *P. olivaceus* shared many features with that of other fishes and mammals. Homology search was performed using BLASTP, and the results revealed that *P. olivaceus* GATA6 protein is highly similar to other known GATA6 proteins, including the proteins in *Stegastes partitus* (94%), *Takifugu rubripes* (92%), *C. Semilaevis* (90%), *Oryzias latipes* (88%) and *Mus musculus* (52%).

**Figure 4:**
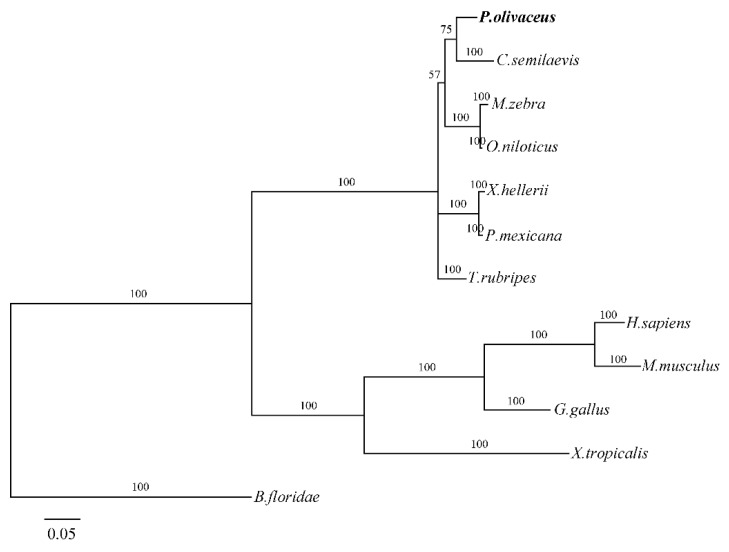
Phylogenetic tree showing the relationship of *P. olivaceus* GATA6 with the GATA6 of other vertebrates. A phylogram was constructed using MrBayes (mcmc = 200,000 generations). The species and GenBank accession numbers or Ensembl numbers are as follows: *H. sapiens*, NP_005248.2; *M. musculus*, NP_034388.2; *G. gallus*, NP_990751.1; *X. tropicalis*, NP_989422.1; *C. semilaevis*, XP_008332620.1; *M. zebra*, XP_004542926.1; *O. niloticus*, XP_005476517.1; *X. hellerii*, XP_005809506.1; *P. Mexicana*, XP_014853786.1; *T. rubripes*, XP_011614478.1; *B. floridae*, ACR66216.1. mcmc, Markov Chain Monte Carlo analysis.

To study the relationship between *P. olivaceus* and other vertebrates, we constructed a phylogenetic tree by using MrBayes based on the deduced amino acid sequences of GATA6 (Figure 4). In the phylogenetic tree, *P. olivaceus* and other teleosts were clustered into one group with 100% bootstrap support, and other higher tetrapods were clustered in another group with 100% bootstrap support. The two groups with 100% bootstrap support were subsequently merged into a cluster.

### 2.3. Genomic Synteny Analysis of GATA6

**Figure 5:**
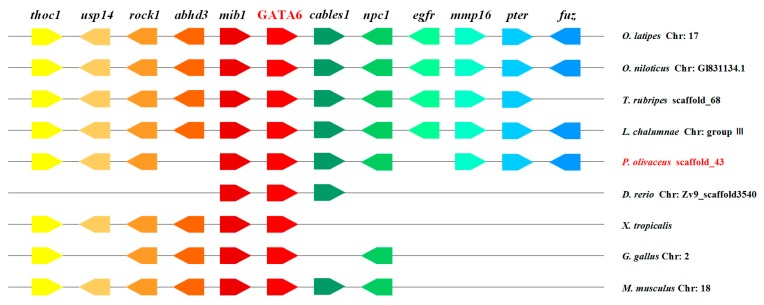
Chromosome synteny of GATA6 of *P. olivaceus* with that of other vertebrates. The pentagons with different colors represent various genes and their respective direction. Gene order was determined according to the relative position of genes in the same chromosome or scaffold. Chr, chromosome.

The location of GATA6 in many vertebrates was determined based on their published whole-genome sequences of these organisms. *P. olivaceus* GATA6 was located on scaffold_43 based on the result of the whole-genome sequence of *P. olivaceus* (unpublished). A conserved syntenic relationship was observed between the *GATA6* gene and its surrounding genes in vertebrates ranging from fish to tetrapods (Figure 5). The *GATA6* gene is generally flanked by *mib1* and *cables1*, and the surrounding genes of GATA6 were conserved in *P. olivaceus* compared with those in other teleosts, although a few of the genes were lost or shifted. In summary, the upstream and downstream genes of GATA6 exhibited conserved synteny in spite of the number of changes (loss or shift) that occurred during evolution.

### 2.4. Expression Patterns of GATA6 at Different Embryonic Developmental Stages and in Different Tissues

**Figure 6:**
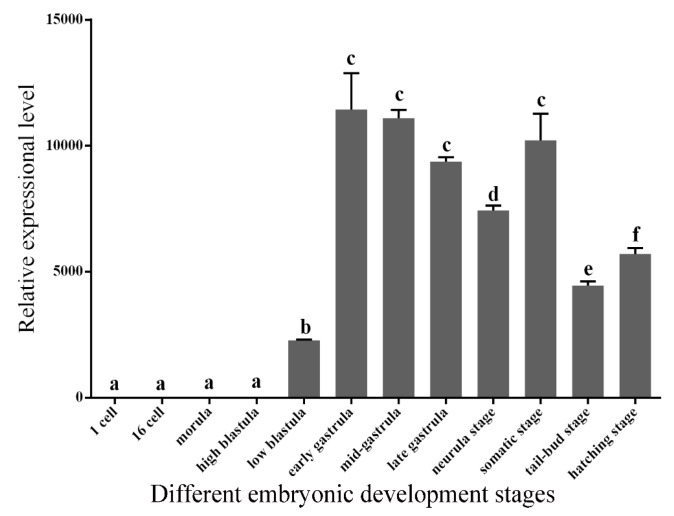
Relative expression of GATA6 during embryonic development from fertilized egg to the hatching stage in *P. olivaceus*. qRT-PCR analysis was used to quantify *GATA6* mRNA with *18sRNA* as a reference. The relative expression variance is presented as a ratio (amount of *GATA6* mRNA normalized to the values of the corresponding reference genes). The data are shown as the mean ± SD (*n* = 3). Columns with different letters show a significant difference (*p* < 0.05).

The temporal expression pattern of *P. olivaceus* GATA6 at the early stage of embryonic development was investigated via qRT-PCR. The result showed that *P. olivaceus GATA6* mRNA were nearly undetectable in the stage prior to the low blastula stage. As embryonic development progressed, mRNA expression of *P. olivaceus* GATA6 significantly increased during blastula stages and showed the highest levels at the gastrula and somatic stages. After the gastrula stage, the relative mRNA expression of *P. olivaceus* GATA6 remained stable until the hatching stage (Figure 6).

**Figure 7:**
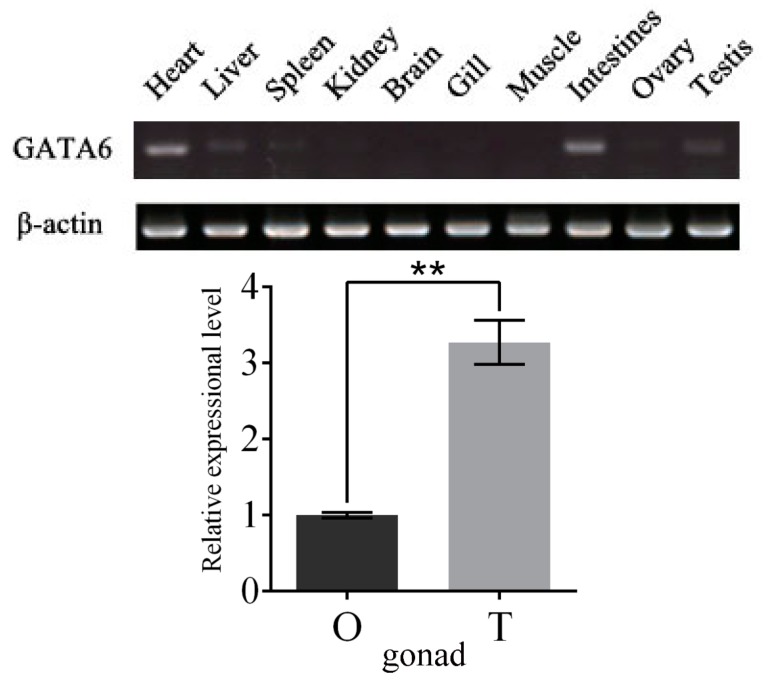
(**A**) Analysis of GATA6 transcripts for various tissues in *P. olivaceus*. Semi-quantitative RT-PCR analysis was used to quantify *GATA6* mRNA with *β-actin* as a reference; and (**B**) analysis of GATA6 transcripts in the gonads of *P. olivaceus*. qRT-PCR analysis was used to quantify *GATA6* mRNA with *β-actin* as a reference. The relative expression variance is presented as a ratio (amount of *GATA6* mRNA normalized to the values of the corresponding reference genes). The data are shown as the mean ± SD (*n* = 3). The asterisks show a significant difference (*p* < 0.01).

The distribution pattern of *P. olivaceus* GATA6 in different tissues was detected by semi-quantitative RT-PCR. As shown in Figure 7A, *P. olivaceus* GATA6 transcripts were highly expressed in heart, intestines, liver and testis, but weakly expressed in spleen, kidney, gill and ovary; the transcripts were nearly undetectable in brain and muscle. To further verify the differences in mRNA expression of *P. olivaceus* GATA6 between ovary and testis, we detected the relative expression of GATA6 by using qRT-PCR. The results showed that GATA6 expression was significantly higher in the testis than ovary (Figure 7B).

### 2.5. In Situ Hybridization (ISH) of P. olivaceus GATA6 in Gonadal Sections

**Figure 8:**
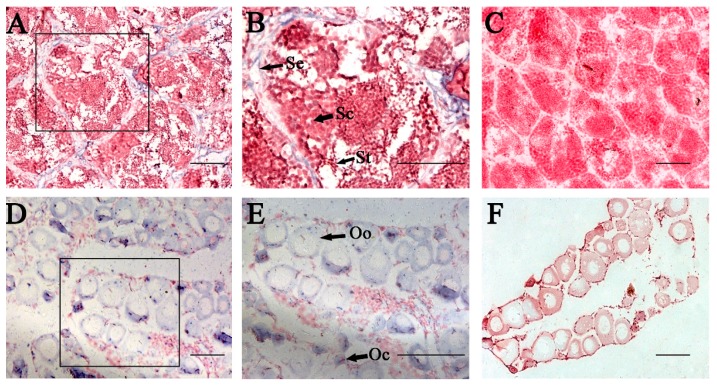
Analysis of mRNA expression of *P. olivaceus* GATA6 in adult gonad by using in situ hybridization (ISH). The positive cells (**A**,**B**,**D**,**E**) were stained with purple or blue, whereas the negative control with sense probe hybridization (**C**,**F**) was unstained. *P. olivaeus* GATA6 mRNA were observed in Sertoli cells (**B**), primary and secondary oocytes and oogonia (**E**). This experiment was done five times. Abbreviations: Se, Sertoli cells; Sc, spermatocytes; St, spermatids; Oo, oogonia; Oc, oocytes. Scale bars represent 100 μm.

The distribution of *P. olivaceus GATA6* mRNA in gonadal sections was analyzed through ISH (Figure 8). Histological observation of the testis was performed at the telophase of Stage III; in this stage, the testes mainly consist of primary and secondary spermatocytes, and spermatogonia are sporadically situated adjacent to the interstitial compartment and at the intertubular location of the spermatogenic tubules (Figure 8C). *GATA6* mRNA signals were found only in Sertoli cells (Figure 8A,B). Histological observation of the ovary was performed at the telophase of Stage II; in this stage, the ovary mainly consists of oocytes in Stages II and III (Figure 8F). mRNA signals of GATA6 were distributed throughout the cytoplasm of primary and secondary oocytes and in oogonia (Figure 8D,E). Hybridization signals were not observed in the gonadal sections by using the respective sense probes (Figure 8C,F).

### 2.6. Expression Levels of GATA6 and CYP19A1 in Cultured P. olivaceus Testis Cells Stimulated with 17α-MT

As described above, GATA6 expression was significantly high in the testis, and its mRNA signals were found only in Sertoli cells. A study has reported that the *P. olivaceus* genetically female larvae could be induced to sex-reversal after being treated with 17α-MT, which is the most preferred hormone used to induce masculinization [20]. Furthermore, 17α-MT treatment suppresses aromatase CYP19A1, which is critical for ovary development [21]. By analyzing the promoter of CYP19A1, we found a recognition sequence for *P. olivaceus* GATA6. Therefore, we wanted to know whether the expression levels of *P. olivaceus* CYP19A1 and GATA6 in testis cells can be altered by 17α-MT. Additionally, we aimed to figure out the role of GATA6 in testis development.

**Figure 9:**
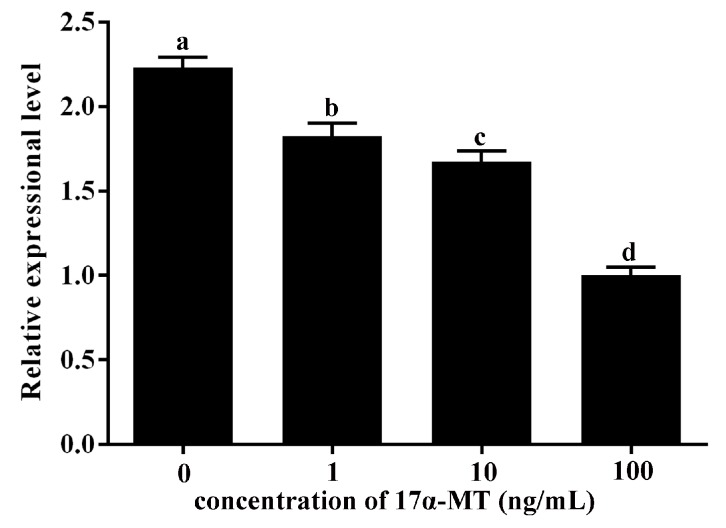
Expression of CYP19A1 mRNA in *P. olivaceus* testis cells stimulated with 1, 10 and 100 ng/mL 17α-MT. qRT-PCR analysis was used to quantify CYP19A1 mRNA with *β-actin* as a reference. The relative expression variance is presented as a ratio (amount of CYP19A1 mRNA normalized to the values of the corresponding reference genes). The data are shown as the mean ± SD (*n* = 3). Columns with different letters show a significant difference (*p* < 0.05).

**Figure 10:**
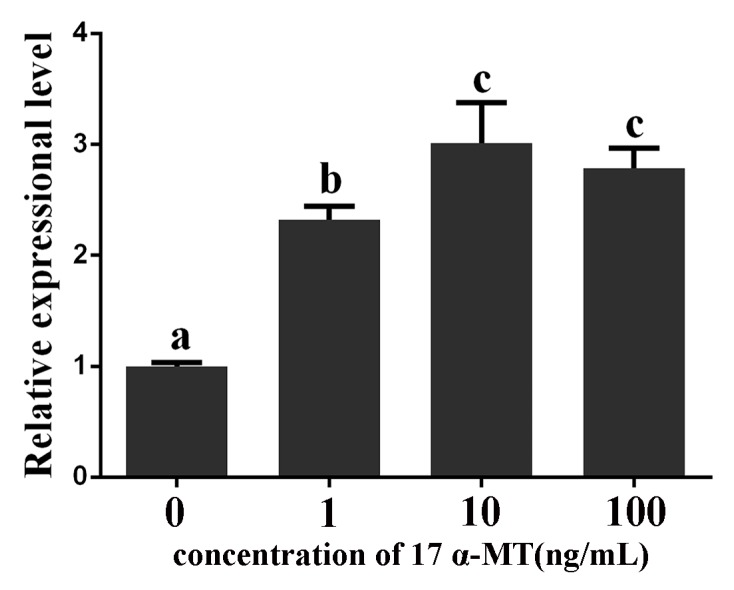
Expression of GATA6 mRNA in *P. olivaceus* testis cells stimulated with 1, 10 and 100 ng/mL 17α-MT. qRT-PCR analysis was used to quantify GATA6 mRNA with *β-actin* as a reference. The relative expression variance is presented as a ratio (amount of GATA6 mRNA normalized to the values of the corresponding reference genes). The data are shown as the mean ± SD (*n* = 3). Columns with different letters show a significant difference (*p* < 0.05).

The qRT-PCR results showed that stimulation of *P. olivaceus* testis cells with 17α-MT reduced the amount of CYP19A1 transcripts, and CYP19A1 expression was downregulated with the increase of stimulation concentration (Figure 9). However, expression of GATA6 increased, and GATA6 expression was upregulated with the increase of stimulation concentration (Figure 10).

## 3. Discussion

Sex differentiation is the process wherein the genital ridge differentiates into either testis or ovary. Studies have recently shown that some transcription factors, such as SOX9 and GATA4, participate in sex differentiation and gonadal development [22,23]. GATA transcription factors regulate the development and differentiation of eukaryotic organisms. GATA6, a member of the GATA family, is associated with gonadal sex differentiation and development and regulates the development of the heart, liver and intestine [19,24,25].

### 3.1. P. olivaceus GATA6 Forms the L-Type Protein

In this study, we obtain the full-length cDNA of *P. olivaceus* GATA6. The coding sequence is 1608 bp long and encodes 535 amino acids. The *P. olivaceus* GATA6 protein contains two typical zinc finger domains, which are highly similar to GATA6 of other vertebrates GATA6 (Figure 2). Thus, *P. olivaceus* GATA6 can identify and bind to the consensus DNA motif (A/T)GATA(A/G), thereby regulating the transcription of target genes [1]. In mammals, the mRNA of GATA6 uses two Met codons in frame as transcriptional initiation and produces L- and S-type GATA6 through leaky ribosome scanning [26,27]. Recent studies have revealed that L-type GATA6 is potentially more active than the S-type GATA6 [26,28]. Human and mouse GATA6 form the L-type protein, and S-type GATA6 is present in *Gallus gallus* and *Anser cygnoides* [29]. On the basis of the multiple amino acid sequence alignment of GATA6, we found that *P. olivaceus* GATA6 forms the L-type protein, which is similar to *C. semilaevis* GATA6 [18]. To date, the functional significance of L- and S-type GATA6 still needs to be investigated.

The phylogenetic tree shows that GATA6 of all vertebrates are clustered in one group and then separated into two subgroups. *P. olivaceus* GATA6 is clustered with the teleost GATA6 subgroup while the tetrapods GATA6 were clustered in another subgroup. Phylogenetic analysis demonstrates that *P. olivaceus* GATA6 is clustered with its homolog from *C. semilaevis*, which also belongs to Pleuronectiformes [18,30]. Based on the protein sequences, the conserved and characteristic domains and the phylogenetic analysis, the sequence we obtained is confirmed to be *P. olivaceus* GATA6.

### 3.2. P. olivaceus GATA6 Is Highly Conserved in Vertebrates

GATA6 has been studied in several vertebrates, including human, mouse, porcine and tongue sole [18,31,32]. Moreover, GATA6 is annotated in published genomes of other model organisms. We compared the gene structure of *P. olivaceus* GATA6 with those of GATA6 in other vertebrates (Figure 3). Based on the number of exons, GATA6 is highly conserved in primates, rodents, birds and amphibians. Interestingly, GATA6 of teleosts are classified into two kinds based on the number of exons. GATA6 of *P. olivaceus*, *Oreochromis niloticus* and *Maylandia zebra* contain eight exons, whereas the GATA6 of other teleosts contain seven exons, similar to tetrapods. We speculate that the extra exon has been lost during the evolution of GATA6 from teleosts to tetrapods [33].

Chromosome synteny of the GATA6 gene was analyzed among fish, frog, chicken and mouse genomes (Figure 5). The results reveal that the location of *P. olivaceus* GATA6 is highly similar to other teleosts, but different from frog, chicken and mouse. This difference may be attributed to gene deletion or rearrangement during evolution. Based on these results, we speculate that *P. olivaceus* GATA6 is conserved during evolution and performs functions similar to those of GATA6 in other vertebrates.

### 3.3. P. olivaceus GATA6 Regulates Organs Originating from Endoderm and Mesoderm

The temporal expression profile of *P. olivaceus* GATA6 during embryonic development shows that the transcripts are very low starting from the unfertilized eggs to the high blastula stage, although it begins to increase at the blastula stage. This result is consistent with the result for other GATA members in teleosts [18,30]. Endoderm differentiation and organogenesis begin at the blastula stage. Moreover, studies have demonstrated that GATA6 is essential for endoderm differentiation [24,34,35,36]. Our results suggest that *P. olivaceus* GATA6 regulates the organs originating from endoderm and mesoderm.

### 3.4. P. olivaceus GATA6 Transcripts Are More Abundant in Testis than Ovary

The tissue distribution pattern of *P. olivaceus* GATA6 shows that GATA6 is expressed in the heart, intestines, liver, spleen, kidney and gonad, similar to the expression distribution of GATA6 of other vertebrates [5,6]. Furthermore, the amount of *P. olivaceus* GATA6 transcripts is significantly higher in testis than in ovary according to the qRT-PCR result (Figure 7A,B). Given the sexually dimorphic pattern of GATA6 expression, it is possible that *P. olivaceus* GATA6 influences gonadal development by binding to target genes related to sex determination.

### 3.5. P. olivaceus GATA6 Is Expressed in Oogonia, Oocytes and Sertoli Cells

In human and mouse, GATA6 are only expressed in Sertoli and Leydig cells, which are classified as somatic cells [13,37]. By contrast, *C. semilaevis* GATA6 mRNA is expressed in spermatogonia, spermatocytes and Sertoli cells [18]. In the present study, the ISH analysis of testis shows that the *P. olivaceus* GATA6 transcripts were only detected in Sertoli cell. This result is similar to that for mammalian GATA6, suggesting that the functions of GATA6 may be analogical in testis. In mammalian ovaries, GATA6 are strongly expressed in corpora lutea and follicles [10,31,38]. In addition, the loss of GATA4 and GATA6 using knockout technology can lead to abnormal ovarian structure and infertility, including oocyte reduction and ovulation failure [14]. Compared with mammalian gonads, ovaries of teleosts do not have corpus luteum and follicles, whose structure is relatively uncomplicated. Our ISH analysis of ovary shows that the *P. olivaceus* GATA6 mRNA is expressed in oocytes and oogonia. Therefore, we speculate that GATA6 is necessary to maintain gonad function and oogenesis.

### 3.6. P. olivaceus GATA6 Is Possibly Involved in the Synthesis of 17β Estradiol by Regulating CYP19A1

In mammals, GATA6 expression is hormonally controlled [16,37]. Our study found that stimulation with 17α-MT can enhance GATA6 expression level in *P. olivaceus* testis cells (Figure 9), indicating that *P. olivaceus* GATA6 is also hormonally controlled.

The combined loss of GATA4 and GATA6 could disrupt the function of gonads [14,15]. Aromatase CYP19A1 is the key enzyme in estrogen synthesis. Treatment with aromatase inhibitor can induce a full functional sex reversal in female adult gonochoristic teleosts, such as *O. latipes*, *O. niloticus* [39,40] and *Danio rerio* [41]. Thus, GATA6 and CYP19A1 are both critical in gonad development. In addition, *P. olivaceus* CYP19A1 is possibly a target gene of GATA6 given that there is a recognition sequence for GATA6 in the CYP19A1 promoter. Stimulation with 17α-MT could improve the GATA6 expression level, but reduced the CYP19A1 expression level in *P. olivaceus* testis cells (Figure 9 and Figure 10). Our results strongly imply that *P. olivaceus* GATA6 protein may exert a negative regulatory effect on transcription of *P. olivaceus* CYP19A1 or it may increase as part of a mechanism activated to compensate the decrease of estradiol. A comparison of previous and present results suggests that GATA6 in *P. olivaceus* may play an important role in gonadal development and reproduction.

## 4. Materials and Methods

### 4.1. Ethics Statement

This research was conducted in accordance with the Institutional Animal Care and Use Committee of the Ocean University of China and the China Government Principles for the Utilization and Care of Vertebrate Animals Used in Testing, Research and Training (State Science and Technology Commission of the People’s Republic of China for No. 2, 31 October 1988.).

### 4.2. Specimen and Tissue Collection

*P. olivaceus* specimens used in this study were obtained from a fish farm in Yantai, Shandong Province, China, and maintained at 17 °C. For the experiments on gene expression, tissues and organs were collected from three 1-year-old male and female *P. olivaceus* individuals. Samples were snap-frozen in liquid nitrogen and then stored at −80 °C until further use. Each sample was collected in triplicate.

Fertilized eggs were obtained through artificial fertilization and incubated at 17 ± 1 °C in sterile sea water with an open recirculation water system and sufficient air supply. Embryos at different developmental stages were observed under a stereomicroscope. Three pools of samples at 12 embryonic stages (1-cell, 4-cell, 16-cell, morula, high blastula, low blastula, early gastrula, mid-gastrula, late gastrula, neurula, tail bud and hatching stages) were separately collected from mixed families by using a nylon net (100-mesh). Embryos were immersed in 1 mL of RNAwait liquid (Solarbio, Shanghai, China) overnight at 4 °C and then stored at −80 °C until further use.

The gonads (ovary and testis) used for ISH were washed with phosphate buffer saline and then fixed in 4% paraformaldehyde in PBS overnight at 4 °C. After removing the envelopes, the gonads were dehydrated in a gradient series of increasing methanol concentration and stored in 100% methanol at −20 °C.

### 4.3. RNA Extraction and cDNA Synthesis

Total RNA was extracted using TRIzol Reagent (Invitrogen, Carlsbad, CA, USA) according to the manufacturer’s protocol. The extracted total RNA was treated with RNase-free DNase I (TaKaRa, Dalian, China) to remove DNA contaminants and then frozen at −80 °C. Reverse transcription and cDNA synthesis were performed with 1 μg of total RNA and random hexamer primers by using a Reverse Transcriptase M-MLV Kit (TaKaRa) according to the manufacturer’s protocol. The quality and quantity of the total RNA were evaluated electrophoretically using 1.5% agarose gel and spectrophotometrically using Nano Photometer Pearl (Thermo Scientific, Carlsbad, CA, USA).

### 4.4. Molecular Cloning of GATA6

A pair of degenerate primers (Table 1) was designed based on the conserved sequences of GATA6 in other teleosts. PCR amplification was performed as follows: initial denaturation step at 95 °C for 5 min; 30 cycles at 95 °C for 30 s, 55 °C for 30 s and 72 °C for 1 min; and a final extension at 72 °C for 10 min. 5′ and 3′ rapid amplification of cDNA ends (RACE) was performed to isolate the full-length cDNA of GATA6 from gastrula using a SMART RACE cDNA Amplification Kit (Clontech, Beijing, China) in accordance with the manufacturer’s protocol. Gene-specific primers (GSPs) were designed based on the known cDNA sequence. The GSPs used in nested PCR were 5′ RACE1 and 5′ RACE2 for 5′ RACE (Table 1) and 3′ RACE1 and 3′ RACE2 for 3′ RACE (Table 1). PCR was conducted according to the SMART RACE amplification methods. PCR products were separated through 1.5% agarose gel electrophoresis, purified using a Zymoclean Gel DNA Recovery Kit (Zymo Research, Orange, CA, USA), cloned into the pMD-18T vector (TaKaRa) and sequenced.

### 4.5. Semi-Quantitative RT-PCR and qRT-PCR

Specific primer pairs (GATA6-RT-FW/RV and CYP19A1-RT-FW/RV, Table 1) used in RT-PCR were designed based on the characteristics of GATA6 and CYP19A1. Pre-experiments were conducted to confirm the generation of single cDNA PCR products.

Total RNA was extracted from embryos and adult tissues, followed by cDNA synthesis. Three biological replicates of each sample were analyzed, and each sample was run in triplicate. Semi-quantitative RT-PCR and qRT-PCR were performed according to the methods described by Liu et al. [42] and Liu et al. [18], respectively.

### 4.6. ISH

The ISH of GATA6 expression in gonads was performed using the probe (Table 1) spanning the 3′-UTR of GATA6 cDNA. DIG-labeled RNA sense and antisense probes were synthesized using a DIG RNA Labeling Kit (SP6/T7) (Roche, Mannheim, Germany) according to the manufacturer’s protocol. ISH on paraffin sections of gonads was performed according to the method described by Gao et al. [43].

### 4.7. Hormonal Stimulation and Primary Cultures of P. olivaceus Testis Cells

Testes of *P. olivaceus* were collected, washed and minced to isolate cells. The minced tissue was transferred into culture flasks containing DMEM/F12 medium and incubated for 8 h at 24 °C. The consumed medium was replaced with a fresh one. The cells were subsequently cultured in DMEM/F12 medium containing 20% FBS, 10 mM HEPES, 10 mM non-essential amino acids, 2 mM l-glutamine, 50 μg/mL streptomycin and 50 U/mL penicillin in air. The reagents and media were purchased from Gibco (Thermo Fisher Scientific Inc.).

The *P. olivaceus* testis cells were separately stimulated with 1, 10 and 100 ng/mL 17α methyl testosterone (17α-MT). After 24 h, total RNA was extracted and qRT-PCR was performed.

### 4.8. Bioinformatics Analysis and Phylogenetic Tree Construction

Homologous nucleotide and protein sequences were confirmed through BLAST searches in NCBI and Ensembl. Multiple sequence alignments were conducted using ClustalX 2.1. A phylogenetic tree was constructed using MrBayes 3.2.3.

### 4.9. Statistical Analysis

qRT-PCR data were statistically analyzed using one-way ANOVA followed by Fisher’s Least Significant Difference (LSD) test using SPSS 20.0 (IBM, New York, NY, USA); *p* < 0.05 indicated statistical significance.

## 5. Conclusions

We report on the full-length cDNA sequence and genomic organization of *P. olivaceus* GATA6. The result of bioinformatics analysis showed that GATA6 is conserved in terms of genomic structure and potential regulatory domains. The semi-RT-PCR result shows that *P. olivaceus* GATA6 mRNA is detected in heart, liver, intestines and gonads, which all originate from the mesoderm and endoderm. Furthermore, *P. olivaceus* GATA6 shows a sexual expression dimorphic pattern in gonads, and its expression was significantly higher in testis than in ovary. The ISH analysis of gonads shows that *P. olivaceus* GATA6 mRNA is detected in oocytes, oogonia and Sertoli cells. In our study, 17α-MT treatment moderately upregulated *P. olivaceus* GATA6, but downregulated CYP19A1 in testis cells. We speculate based on the finding that *P. olivaceus* GATA6 may be involved in 17β estradiol synthesis by regulating CYP19A1. These results suggest that *P. olivaceus* GATA6 may perform an essential function in gonadal development.

## Figures and Tables

**Table 1:** Primers used in this study. RACE, rapid amplification of cDNA ends.

Primers	Sequence (5′–3′)	*T*m (°C)	Usage
*gata6*-Fw	GRTCAAGSGAGAAGTATCC	59	Degenerate PCR
*gata6*-Rv	CWGATRGCTGGGTCAAG	59	Degenerate PCR
*gata6*-5′Rv1	GCTGGATACTTCTCGCTTGAC	63	5′ RACE
*gata6*-5′Rv2	CCGTGGTTGGTGGTCATTAT	62	5′ RACE
*gata6*-3′Fw1	CGCTCGCCATGAAGAAAGA	62	3′ RACE
*gata6*-3′Fw2	ACAAAGGGATCCTCTGGAAATAA	62	3′ RACE
*gata6*-RT-Fw	CATCCACCTCCTCATCCAATTC	62	qRT-PCR/semi-RT-PCR
*gata6*-RT-Rv	CATCTGATGGCTGGGTCAAG	62	qRT-PCR/semi-RT-PCR
*Cyp19a1*-RT-Rv	CACCTTTCTGTTTGGGTTTG	62	qRT-PCR
*Cyp19a1*-RT-Rv	GTCTCCATACCTCTTGTTGTAG	62	qRT-PCR
*gata6*-ISH-Fw	CATCCACCTCCTCATCCAATTC	62	ISH probe
*gata6*-ISH-Rv	CCACACCAATCTGGAAACAAATC	62	ISH probe
